# Identification of Variants in Primary and Recurrent Glioblastoma Using a Cancer-Specific Gene Panel and Whole Exome Sequencing

**DOI:** 10.1371/journal.pone.0124178

**Published:** 2015-05-07

**Authors:** Selene M. Virk, Richard M. Gibson, Miguel E. Quinones-Mateu, Jill S. Barnholtz-Sloan

**Affiliations:** 1 Department of Epidemiology and Biostatistics, Case Western Reserve University, Cleveland, Ohio, USA; 2 University Hospital Translational Laboratory, University Hospitals Case Medical Center, Cleveland, Ohio, USA; 3 Case Comprehensive Cancer Center, Case Western Reserve University, Cleveland, Ohio, USA; 4 Department of Pathology, Case Western Reserve University, Cleveland, Ohio, USA; University of Navarra, SPAIN

## Abstract

Glioblastoma (GBM) is an aggressive, malignant brain tumor typically resulting in death of the patient within one year following diagnosis; and those who survive beyond this point usually present with tumor recurrence within two years (5-year survival is 5%). The genetic heterogeneity of GBM has made the molecular characterization of these tumors an area of great interest and has led to identification of molecular subtypes in GBM. The availability of sequencing platforms that are both fast and economical can further the adoption of tumor sequencing in the clinical environment, potentially leading to identification of clinically actionable genetic targets. In this pilot study, comprised of triplet samples of normal blood, primary tumor, and recurrent tumor samples from three patients; we compared the ability of Illumina whole exome sequencing (ExomeSeq) and the Ion AmpliSeq Comprehensive Cancer Panel (CCP) to identify somatic variants in patient-paired primary and recurrent tumor samples. Thirteen genes were found to harbor variants, the majority of which were exclusive to the ExomeSeq data. Surprisingly, only two variants were identified by both platforms and they were located within the *PTCH1* and *NF1* genes. Although preliminary in nature, this work highlights major differences in variant identification in data generated from the two platforms. Additional studies with larger samples sizes are needed to further explore the differences between these technologies and to enhance our understanding of the clinical utility of panel based platforms in genomic profiling of brain tumors.

## Introduction

Glioblastoma (GBM) is the most frequently occurring primary brain tumor in adults [1]. Although considered a rare cancer, with an incidence rate of 2 to 3 cases per 100,000 people in the United States, this tumor contributes disproportionately to cancer morbidity and mortality [1, 2]. Current standard therapy for GBM patients is surgical resection of the primary tumor followed by adjuvant radiotherapy and chemotherapy (i.e. “Stupp” protocol) [3]; however, in spite of aggressive treatment, GBM recurs in many patients within two years [4]. Because of this high likelihood of recurrence, it is imperative that we obtain a better understanding of the genetic and molecular changes that occur after primary tumor resection and treatment. This information could be vital to the development of new treatments with the potential to increase survival of GBM patients.

The genetic differences between primary and recurrent tumors have yet to be fully characterized, which have limited the development of effective targeted therapies to combat recurrent GBM [5]. Mutations in specific genes have been shown to be prevalent in GBM cases [6, 7]. Due to known inter- and intra- individual heterogeneity in GBM [8, 9], considerable effort has been made to not only identify individually mutated genes but to develop classification schemes to characterize the tumors by molecular subtype [10, 11]. The end goal is to use tumor classification as a molecular diagnostic tool or a prognostic indicator of overall survival, ultimately leading to discovery of new treatment strategies.

Deep (next generation) sequencing is revolutionizing our understanding of somatic changes occurring in the cancer genome. Studies comparing multiple tumor types are shedding light on both the relationship between genetic mutations and cancer type, as well as dysregulated pathways that are common across cancers or specific to subset of cancer types [12–14]. Although the Illumina (Illumina, San Diego, CA) deep sequencing platform is the current leader in clinical cancer research [15], other technologies such as Ion Torrent (Life Technologies, Carlsbad, CA) [16] are suitable for broader screening methods based on reduced sequencing time and cost per sample [17]. In this study we evaluated the ability of the Ion AmpliSeq Comprehensive Cancer Panel (Life Technologies, Carlsbad, CA) to identify variants in triplicate samples (matched blood, primary and recurrent tumors) from three GBM patients and compared the results to whole exome sequencing data obtained using the Illumina platform from the Cancer Genome Atlas (TCGA) project [18].

## Materials and Methods

### Study Patients and Sample Processing

**Fig 1:**
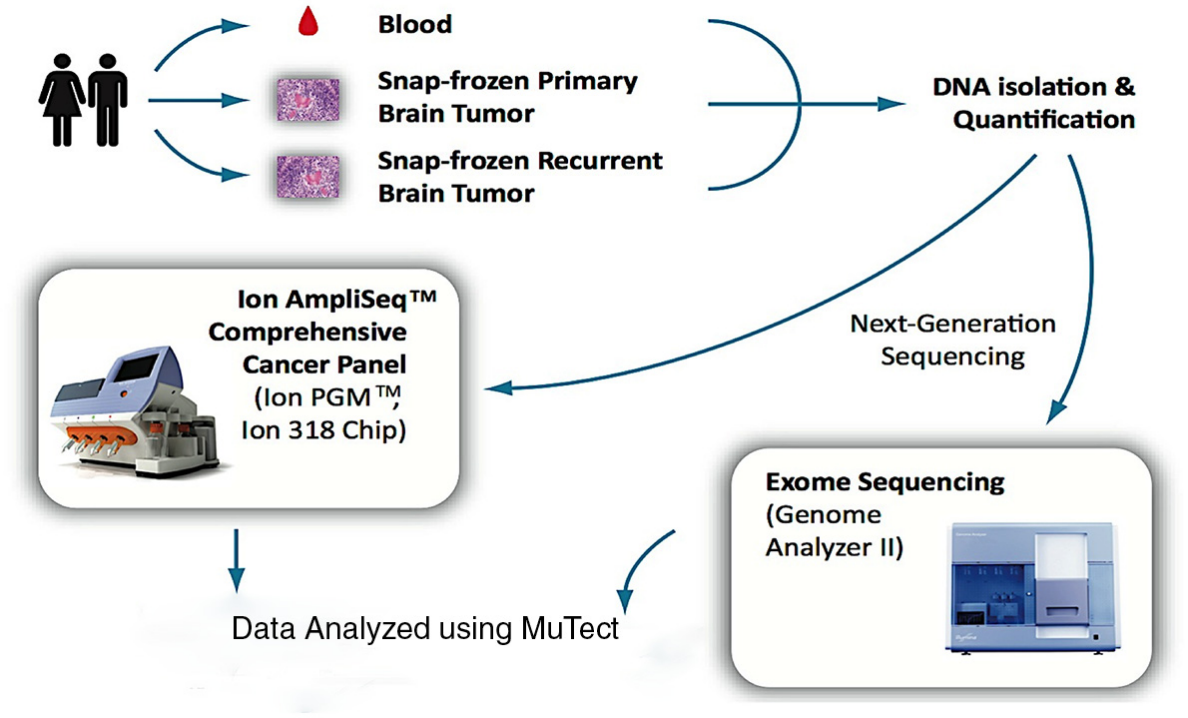
Overview of patient samples and sequencing methods. Sequencing for the AmpliSeq CCP samples was done using the Ion 318 Chip and whole exome sequencing was preformed using the Genome Analyzer II or HiSeq 2000, both from Illumina.

GBM patients were prospectively recruited as part of the ongoing Ohio Brain Tumor Study (OBTS), a prospective study of primary benign and malignant brain tumor patients from the four major academic centers in the state of Ohio. Written consent was received from all patients during their enrollment into OBTS, which occurred prior to specimen collection. All human subjects protocols and procedures were approved by the Institutional Review Board at University Hospitals Case Medical Center, Cleveland, Ohio. Pre-treatment blood samples along with primary and recurrent snap-frozen tumor tissues (triplet samples) were collected from three adult patients. Fig 1 provides an overview of procedures used in this study. Tissues were frozen within 15 to 30 minutes following resection. Samples and clinical information were collected and archived in accordance with guidelines established by The Cancer Genome Atlas [18] and tumor purity was accessed by histological review by board certified neuropathologist. Two sections were taken from each patient sample (3 samples/patient X 2) and one collection of triplet samples from all three patients were sequenced on each of the two sequencing platforms.

Blood was drawn into PAXgene DNA tubes and DNA extracted using PAXgene Blood DNA kit (Qiagen, Valencia, CA). Total DNA was isolated from the snap-frozen brain tumor samples using the Maxwell 16 DNA Purification kit (Promega, Madison, WI). DNA was quantified using Nanodrop ND-100 (Thermo Scientific, Waltham, MA) and Qubit 2.0 Fluorometer (Life Technologies, Carlsbad, CA). Patient-paired triplet DNA samples from three patients were sequenced using (i) whole exome sequencing on the Illumina GA-IIX or HiSeq 2000 [ExomeSeq] (Fig 1) as part of The Cancer Genome Atlas project as detailed in [18] and (ii) the Ion AmpliSeq Comprehensive Cancer Panel (Ion AmpliSeq CCP, Life Technologies). For this, four amplicon pools per sample covering 409 genes implicated in cancer were quantified (2100 Bioanalyzer DNA 7500, Agilent Technologies), prepared and enriched for sequencing on the Ion Sphere Particles (ISPs) using the Ion OneTouch 200 Template Kit v2 (Life Technologies) in the Ion OneTouch™ 2 System (Life Technologies). Templated ISPs were quantified (Qubit 2.0, Life Technologies) and loaded into an Ion 318 Chip (Life Technologies) to be sequenced on the Ion PGM using the Ion PGM Sequencing 200 Kit v2 (Life Technologies). The average loading efficiency in the Ion Torrent PGM was 88% in all nine Ion 318 chips with an average of 5.9 million reads per sample. Individual samples averaged over 5.8 million mapped sequence reads, with a mean read length of 109 base pairs. The average target coverage in the Ion Torrent PGM was 315x and 377x for blood and tumor samples, respectively. In the case of the TCGA Illumina whole exome sequencing, the average target coverage was 121x and 138x for blood and tumor samples, respectively. Signal processing and base calling of sequence data generated from the Ion Torrent PGM was performed with Torrent Analysis Suite version 3.4.2. High quality reads (a quality score >20) from the FASTQ files generated from the Ion Torrent PGM Server were trimmed of adapter, barcode, and primer sequences prior alignment to the human genome assembly 19 (Hg19) sequence.

### Data Processing and Variant Identification

Prior to variant analysis, FASTQ files generated from both platforms were subjected to several preprocessing steps. FASTQ files were aligned to the Human Genome Reference Consortium build 37 (Hg19) using the Burroughs-Wheeler Alignment algorithm as implemented in the BWA software package v.0.7.2, resulting in the production of BAM files of the aligned reads. Following genome alignment, the Genome Analysis Toolkit v.3.2.2 was used to realign reads around insertion/deletions and recalibrate base quality scores for both ExomeSeq and AmpliSeq data. The ExomeSeq reads were subjected to duplicate marking and removal prior to the realignment and base recalibration steps. BAM files were sorted in coordinate order and BAM index files were generated using Picard v.1.119. Variant calling was performed in aligned reads paired by patient samples, i.e., blood (normal) versus primary or recurrent tumor tissue, using MuTect v.1.1.4 [19] along with DbSNP 138. Variants were annotated with ANNOVAR v.111214 (http://www.openbioinformatics.org/annovar/) and read alignments were visualized using IGV v2.3.2 (http://www.broadinstitute.org/igv/). Variant analysis was restricted to variants occurring in exome regions.

## Results

### Patient demographics and treatment

Among the three patients included this study, the average age was 45 years and the mean overall survival was 441 days (Table 1). All but one patient was male and all were white (one patient also self-identified as Hispanic). Each patient received the same treatment, which consisted of total resection followed by adjuvant temozolomide and external beam radiation. In addition, all patients underwent a second surgery upon recurrence. Purity of primary tumor samples was greater than in recurrent tumor samples, ranging from 80–90% to 65–75%, respectively (Table 1).

**Table 1:** Patient demographics and clinical information.

**Patient Number**	**UH1**	**UH2**	**UH3**
**Gender**	Female	Male	Male
**Age at Diagnosis**	48	51	36
**Race/Ethnicity** ^a^	W/NH	W/NH	W/H
**Year Diagnosis**	2007	2008	2009
**Overall Survival (Days)**	665	141	430
**Progression Time (Days)**	50	81	70
**Days to Second Surgery**	413	81	70
**Tumor Sample Purity (Primary/Recurrent)** ^b^	90%/65%	80%/65%	80%/75%

All patients received total resection, followed by temozolomide and external beam radiation of 6,000 Gray in 30 fractions.

^a^W/NH, white not Hispanic or Latino; W/H, white Hispanic or Latino.

^b^Sample purity determined by histological review.

### Variants identified by both ExomeSeq and AmpliSeq CCP

**Fig 2:**
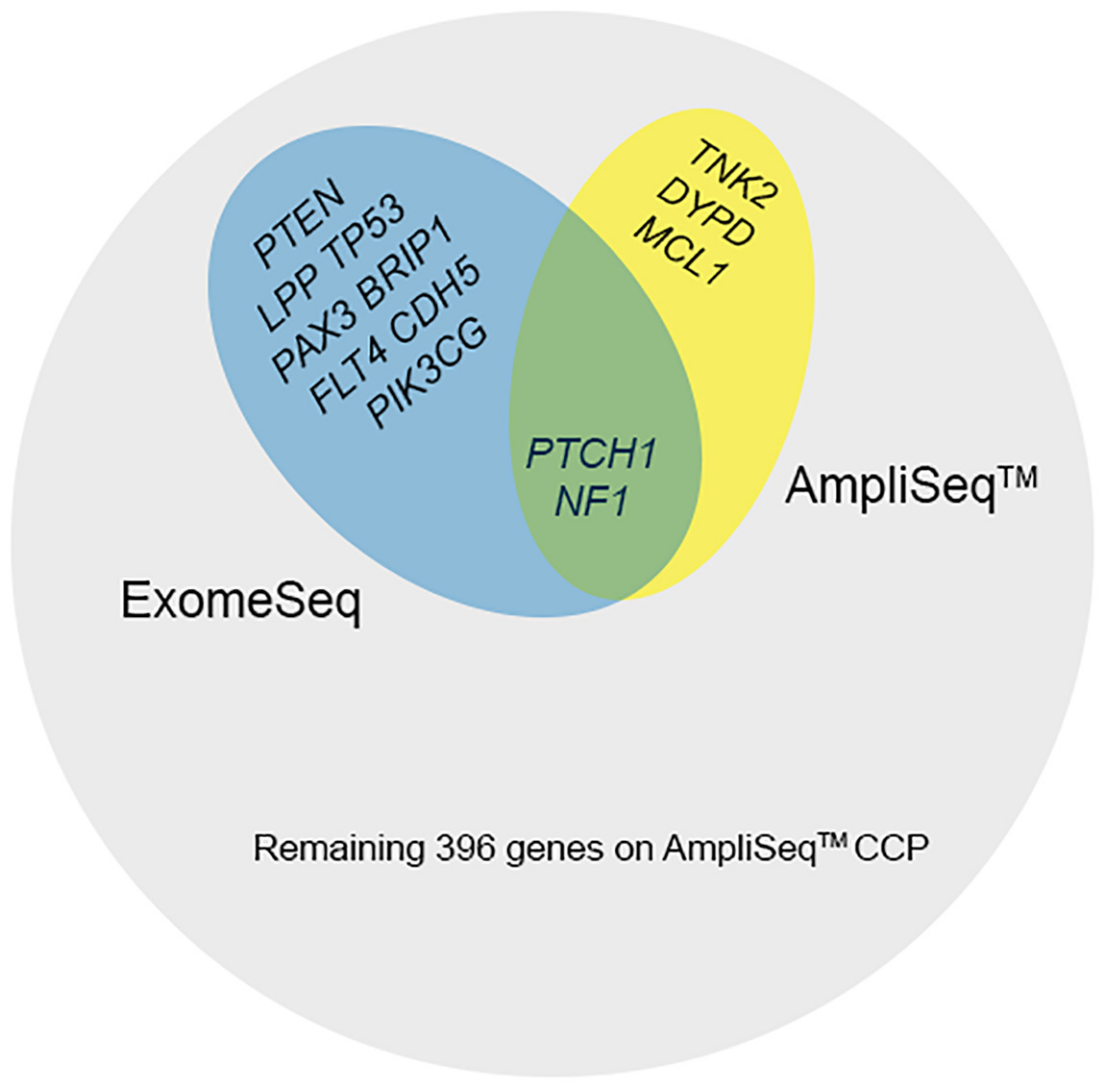
Summary of mutated AmpliSeq CCP genes. Genes containing variants and the associated sequencing platforms are shown. Most variants were associated with ExomeSeq data and there were a total of 409 genes on the AmpliSeq CCP.

Since whole exome sequencing technologies have the capacity to cover all coding genes within the genome, whereas any panel based approach will be limited to a subset of the coding genome, to make the most appropriate comparison between the two technologies we restricted our analysis to the 409 genes included in the AmpliSeq panel. In addition, since we were most interested in variants within coding regions, we also focused on analysis of variants within exons only. Variants were identified in 13 (3%) of the 409 genes analyzed in this study. The majority of variants were found in the ExomeSeq data and only two gene variants were shared between the two platforms (Fig 2).

**Fig 3:**
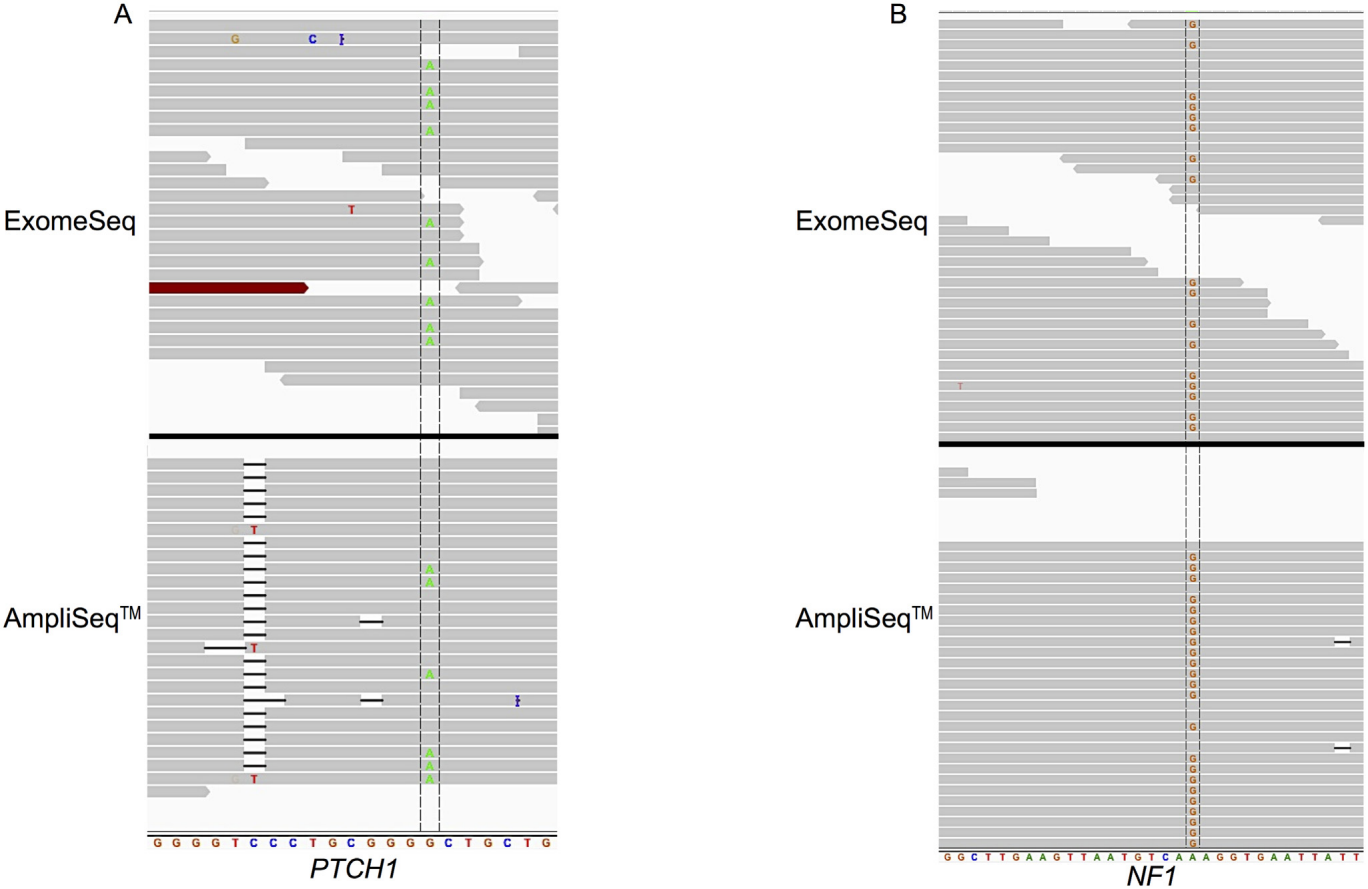
Read alignment view of *PTCH1* (A) and *NF1* (B) genes zoomed in to individual base level. (A) View displayed spans chr9: 98,209,620–98,209,640 of *PTCH1*; variant base is G to A mutation located at base 98,209,634 in primary tumor of patient UH1. (B) View displayed spans chr17: 29,585,500–29,585,530 of *NF1*; variant is G to A mutation located at base 29,585,518 in recurrent tumor of patient UH3. The variant base is located between the two vertical lines crossing the reads.

The variants found by both platforms were in the genes *PTCH1* (patched 1) and *NF1* (neurofibromin 1). Detailed analysis of the *PTCH1* variant revealed similar coverage of the mutated base (Fig 3A) in both the AmpliSeq and ExomeSeq data at 26 and 24 reads, respectively; and the AmpliSeq panel had a lower percentage (23% compared to 38%) of reads containing the alternate base. The other shared variant was found in the *NF1* gene (Fig 3B) and in this case the two platforms differed greatly in terms of read coverage of the mutated base. In the ExomeSeq data, there were 38 reads covering the variant base and 18 (47%) contained the variant; whereas 360 reads in the AmpliSeq data covered the base and 20% (71 reads) contained the variant. *NF1* was also one of the four variants identified in a recurrent tumor sample.

### Variants identified by ExomeSeq but not by AmpliSeq CCP

**Fig 4:**
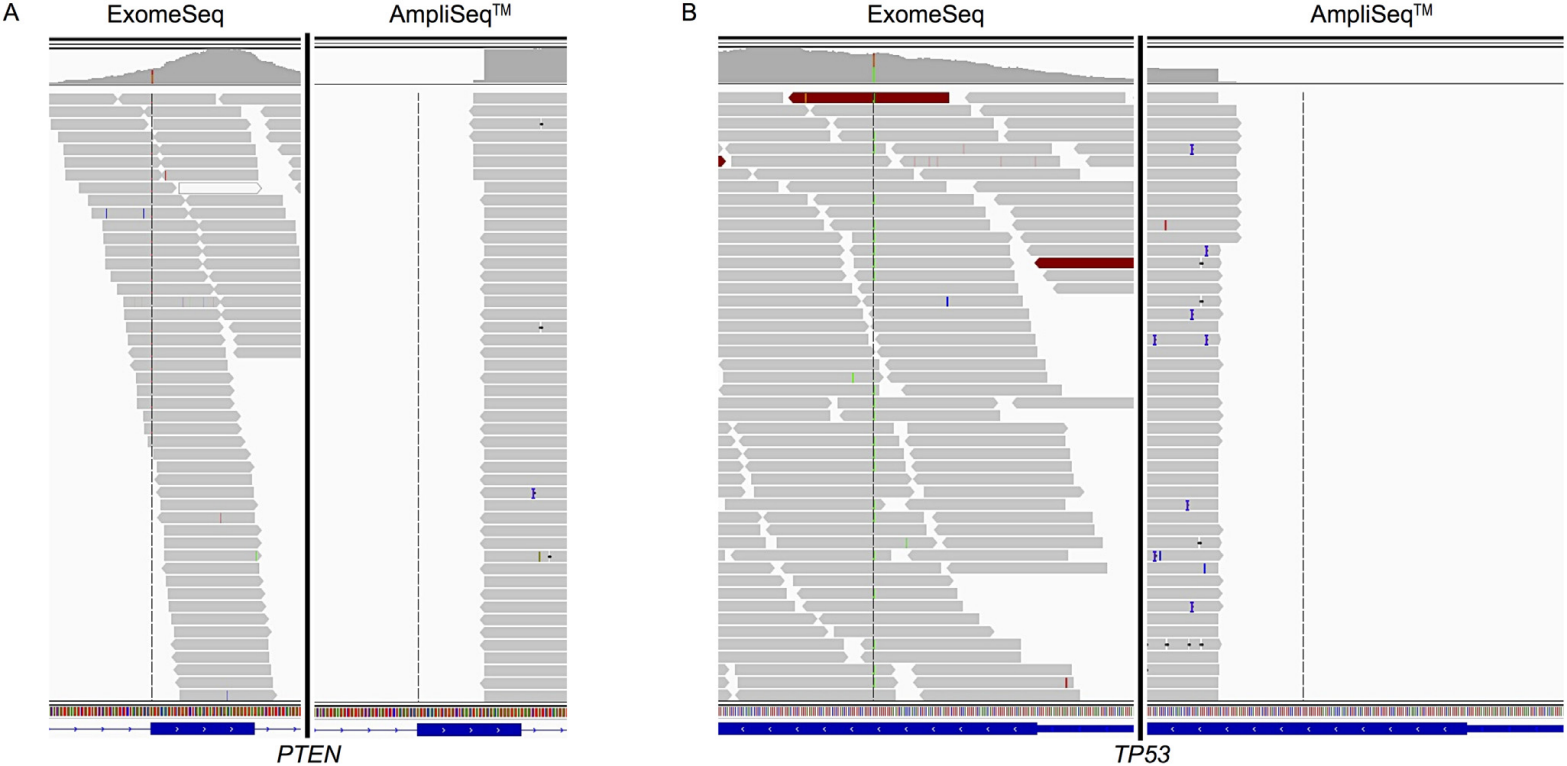
Read alignment view of *PTEN* (A) and *TP53* (B) genes zoomed out to exon level. The AmpliSeq data did not produce any reads covering the variant base in *PTEN*; however, a single read not shown in the graphic did cover the variant base in *TP53* in the recurrent tumor. Data from primary tumors are shown. (A) View displayed spans chr10: 89,653,700–89,653,900 of *PTEN*; variant is T to G mutation located at base 89,653,783. (B) View of *TP53* displays region of chr17: 7,578,400–7,578,600; variant is G to A mutation located at base 7,578,475. The location of the variant base is indicated by the vertical line crossing the reads.

There were a total of 10 variants in eight genes supposedly included in the AmpliSeq CCP that were only identified by the ExomeSeq approach. Two of these genes were *PTEN* (phosphatase and tensin homolog) and *TP53* (tumor protein p53), both of which were found in the primary and recurrent tumors of patient UH3. Both of these genes are frequently mutated in GBM and many other cancers. In the primary tumor, the exon containing the mutated base in *PTEN* was widely covered by the ExomeSeq reads and the mutated base was covered by 26 reads, 65% of which contained the variant; however, the same exon was only partially covered by reads from the AmpliSeq CCP (Fig 4A) none of which covered the mutated base. Similarly, a mutation in the *TP53* gene was covered by 98 ExomeSeq reads, 53% contained the variant base, while no AmpliSeq CCP reads covered this region of the exon (Fig 4B). Analysis of read coverage of *PTEN* and *TP53* in the recurrent tumor of patient UH3 found an increase in the number of reads covering the variant base in comparison to the primary tumor for both genes at 68 and 121, respectively. In terms of the proportion of the reads covering the base that were also mutated, there was a reduction for *PTEN* to 40% and for *TP53*, the proportion was practically the same as in the primary tumor at 54%. In addition, the AmpliSeq data for the recurrent tumor produced a single read covering the mutated base in *TP53*, this read also contained the variant.

The *PIK3CG* (phosphatidylinositol-4,5-bisphosphate 3-kinase, catalytic subunit gamma) gene was the only gene found to be mutated in more than one patient, specifically this gene contained two different variants and one of each was present in the primary tumors of both UH1 and UH3 individuals (Table 2). Interestingly, two variants were identified in the *LLP* (LIM domain containing preferred translocation partner in lipoma) gene, both in the primary tumor of patient UH2 (Table 2). These two variants were located in adjacent bases. Examination of all AmpliSeq CCP genes that contained variants only in ExomeSeq data revealed the *LLP* gene to have the greatest coverage with 1388 and 1403 reads covering the first and second variant, respectively. In both cases, 80% of the reads were mutated.

**Table 2:** Variants identified in Ion AmpliSeq Comprehensive Cancer Panel Genes.

**Patient**	**Tumor Type**	**Gene**	**Chr**	**Location**	**Reference**	**Variant**	**Effect**	**AmpliSeq**	**ExomeSeq**
**UH1**	P	PAX3	2	223084928	G	A	S SNV		x
	P	PIK3CG	7	106508092	C	T	NS SNV		x
	P	PTCH1	9	98209634	G	A	NS SNV	x	x
	P	TNK2	3	195595319	T	G	NS SNV	x	
**UH2**	P	LLP	3	188584016	C	T	NS SNV		x
	P	LLP	3	188584017	C	T	S SNV		x
	P	BRIP1	17	59763203	C	T	NS SNV		x
	P	DYPD	1	98039494	A	G	S SNV	x	
	P	MCL1	1	150551538	C	T	NS SNV	x	
	R	CDH5	16	66420931	G	A	NS SNV		x
**UH3**	P	FLT4	5	180048765	G	A	S SNV		x
	P	PIK3CG	7	106509875	T	A	NS SNV		x
	P	PTEN	10	89653783	T	G	Stopgain		x
	P	TP53	17	7578475	G	A	NS SNV		x
	R	PTEN	10	89653783	T	G	Stopgain		x
	R	TP53	17	7578475	G	A	NS SNV		x
	R	NF1	17	29585518	A	G	NS SNV	x	x

Summary of all variants identified in this study, the majority of which were found in primary tumors. R, recurrent; Chr, chromosome; NS, nonsynonymous; P, primary; SNV, single nucleotide variant.

### Variants identified exclusively by AmpliSeq CCP

In contrast to the variants unique to the ExomeSeq data, three variants in three different genes were identified by the AmpliSeq CCP but not by whole exome sequencing (Fig 2 and Table 2). All three variants were detected in primary tumors of two patients (UH1 & UH2), specifically in the *DPYD* (dihydropyrimidine dehydrogenase), *MCL1* (v-myc myelocytomatosis viral oncogene homolog 1, lung carcinoma derived [avian]), and *TNK2* (tyrosine kinase, non-receptor, 2) genes. None of the three variants unique to AmpliSeq CCP were designated as “covered” by the MuTect software, indicating less than 80% power to detect these variants at 0.3 allelic fraction.

## Discussion

Deep sequencing methodologies have revolutionized many biological and biomedical fields with cancer, particularly diagnostics and treatment strategies, being one of the most impacted [20, 21]. Great effort has been placed on sequencing entire genomes or whole exomes to identify critical cancer-associated mutations that could drive personalized and targeted therapies [22]. Unfortunately, although access to deep sequencing technologies has increased considerably during the last few years, the continued relatively high cost of sequencing and complexity of the analysis has prompted the development of more affordable instruments and methodologies, together with the simplification of the number of genes to be analyzed. The Ion AmpliSeq CCP was developed to sequence all-exon coverage of 409 genes implicated in cancer, that is, over 50% of the Wellcome Trust Sanger Institute Cancer Gene Census (http://cancer.sanger.ac.uk/cosmic/census). In this pilot study we used the AmpliSeq CCP to sequence matched blood, primary and recurrent tumors from three GBM patients and compared the results to whole exome sequencing data obtained using the Illumina platform from The Cancer Genome Atlas (TCGA) project.

Overall, few genes were found to contain variants in this study (13 genes) and among those, only two variants were jointly identified by the two sequencing approaches (ExomeSeq and AmpliSeq CCP). A variant in the *PTCH1* gene was identified in the primary tumor from patient UH1. This tumor suppressor gene functions in the Hedgehog signaling pathway and has been show to be mutated in pediatric medulloblastoma [23]. The second variant was within the gene *NF1* (also a tumor suppressor), which encodes the neurofibromin 1 protein and is a frequently mutated gene in GBM [18]. These two variants were the only two covered by the MuTect software, which effectively makes *PTCH1* and *NF1* the most reliably identified variants in the AmpliSeq CCP data.

There was also little overlap of variants between individual patient and tumor types. The only gene identified as variant in more than one patient was *PIK3CG*, which was mutated in primary tumors from patients UH1 & UH3. This gene contained different variants in the two tumors and was only present in the ExomeSeq data. The genes *PTEN* and *TP53* contained the only gene variants identified in both the primary and recurrent tumors of the same patient, UH3. Although both genes are among those frequently mutated in GBM, these variants were also exclusive to the ExomeSeq data. Further inspection of the alignments at the variant bases revealed only partial coverage of the variant containing exons by the AmpliSeq reads, which is a likely contributor to the variant not being detected by this panel.

It was anticipated that there would be differences in the variants identified from the ExomeSeq and AmpliSeq CCP data. In an effort to limit these differences, we restricted the analysis to only include genes that were on the gene list of the AmpliSeq CCP. The different technological approaches of sequencing an entire exome versus sequencing a focused subset of cancer-related genes, could likely result in finding fewer mutated genes using the more focused approach, as observed in this study. Another point to be mindful of is that intra-tumoral heterogeneity is a characteristic of many cancers, including GBM. The analysis reported here was done using two different cuts from each patient tumor and our observed discrepancies in variant identification could stem from the inherent intra-tumoral heterogeneity in GBM.

This analysis provides evidence that the genes *PTCH1* and *NF1* can be reliably detected in patient samples and that the lack of broad exon coverage can impact variant detection in those regions. The genes included in the AmpliSeq CCP were selected to be representative of a wide selection of cancer types and gene panels more specific for brain and brain tumors may improve variant detection in GBM. Although a few studies have evaluated the use deep sequencing-based panels for rapid cancer genotyping [23, 24], our pilot study demonstrates the potential for the Ion AmpliSeq CCP to contribute to detection of mutations in both basic and clinical cancer research. Further studies, with a larger number of samples will help us understand the differences observed between the two methodologies and add to our current understanding of mutations that drive progression and recurrence in GBM.
